# Establishing cut-points for physical activity classification using triaxial accelerometer in middle-aged recreational marathoners

**DOI:** 10.1371/journal.pone.0202815

**Published:** 2018-08-29

**Authors:** Carlos Hernando, Carla Hernando, Eladio Joaquin Collado, Nayara Panizo, Ignacio Martinez-Navarro, Barbara Hernando

**Affiliations:** 1 Sport Service, Jaume I University, Castellon, Spain; 2 Department of Education, Jaume I University, Castellon, Spain; 3 Department of Mathematics, Carlos III University of Madrid, Madrid, Spain; 4 Faculty of Health Sciences, Jaume I University, Castellon, Spain; 5 Department of Physical Education and Sport, University of Valencia, Valencia, Spain; 6 Department of Medicine, Jaume I University, Castellon, Spain; University of Wisconsin, UNITED STATES

## Abstract

The purpose of this study was to establish GENEA (Gravity Estimator of Normal Everyday Activity) cut-points for discriminating between six relative-intensity activity levels in middle-aged recreational marathoners. Nighty-eight (83 males and 15 females) recreational marathoners, aged 30–45 years, completed a cardiopulmonary exercise test running on a treadmill while wearing a GENEA accelerometer on their non-dominant wrist. The breath-by-breath *V̇*O_2_ data was also collected for criterion measure of physical activity categories (sedentary, light, moderate, vigorous, very vigorous and extremely vigorous). GENEA cut-points for physical activity classification was performed via Receiver Operating Characteristic (ROC) analysis. Spearman’s correlation test was applied to determine the relationship between estimated and measured intensity classifications. Statistical analysis were done for all individuals, and separating samples by sex. The GENEA cut-points established were able to distinguish between all six-relative intensity levels with an excellent classification accuracy (area under the ROC curve (AUC) values between 0.886 and 0.973) for all samples. When samples were separated by sex, AUC values were 0.881–0.973 and 0.924–0.968 for males and females, respectively. The total variance in energy expenditure explained by GENEA accelerometer data was 78.50% for all samples, 78.14% for males, and 83.17% for females. In conclusion, the wrist-worn GENEA accelerometer presents a high capacity of classifying the intensity of physical activity in middle-aged recreational marathoners when examining all samples together, as well as when sample set was separated by sex. This study suggests that the triaxial GENEA accelerometers (worn on the non-dominant wrist) can be used to predict energy expenditure for running activities.

## Introduction

Long-distance races have substantially increased in popularity over recent years by means of both the number of international marathon races, as shown in IAAF calendar (https://www.iaaf.org/competition/calendar), and the number of marathon/ultramarathon finishers constantly raised in the last few decades [1–6]. For example, in 2016, up to fifty marathon races were organized in Spain (http://www.carreraspopulares.com/solomaraton). Since the marathon is one of the most challenging endurance competitions [7,8], runners’ interest for the improvement of training programs and for nutrition advice has been significantly increased in order to improve their marathon time without soreness and preventing energy deficit [9,10]. Elite athletes work closely with multidisciplinary teams (comprising coaches, nutritionists and medical specialists) to prepare training programs in order to achieve their goal [11–13]. Nowadays, recreational athletes are also advised by a wide range of professional experts who analyze training indicators after training sessions, since they are not usually present in each one of them [14,15]. The development of monitoring devices that provide valuable information (i.e. strength parameters, heart rate, movement acceleration, running pace, ground contact time measures, energy consumption, etc.) to athletes, coaches and healthcare experts has been recently targeted in an attempt to improve training session evaluation and design, as well as running performance [16,17].

The use of accelerometers in physical activity evaluation (in terms of intensity, frequency and duration) has exponentially increased since its creation in 1983 [18], being a potential tool to accurately estimate physical activity energy expenditure from accelerometer output data [17,19–21]. Research studies have been focused on the standardization of data collection, wear site, measurement period and data reduction methods, in order to uniformly measure the physical activity across studies [17,21–23]. Additionally, multiple validation researches attempt to distinguish different physical activity categories by cut-point approach [24–27], and to indirectly measure energy cost of physical activity–expressed as Metabolic Equivalent of Task (MET) [17,23,25,28–30]. Therefore, accelerometry may be a useful tool for monitoring athletes.

Among all the accelerometer-based physical activity monitors, the most recent developed triaxial wrist-worn accelerometer, the Gravity Estimator of Normal Everyday Activity (GENEA), has been found to present a high instrument reliability and criterion validity as well as to accurately classify different intensities of physical activity (sedentary, light, moderate and vigorous) [25]. Furthermore, due to its characteristics (watch-like design, small size, light weight and waterproof), the GENEA seems to be one of the most comfortable accelerometer device to wear during free-living condition assessment [24,31]. Previous validation studies of wrist-worn GENEA accelerometer have been performed in different specific populations (adults, children, wheelchair users, pregnant females, etc.) in order to analyze and quantify physical activity in normal daily activities for improvement of lifestyle conditions [22,26,27,29,32–35]. However, this study goes toward the use of accelerometer-based devices to track adult recreational marathoners, a population subset with higher physical and metabolic fitness than standard adult population. The reason of creating cut-points specific to a particular population is because the individualized energy expenditure may fluctuate according to the body weight and composition, sex, age, physical fitness, mechanical efficiency and the environmental conditions under which the activity is performed [16,17,20,36].

Therefore, the main purpose of this study was to establish wrist-worn GENEA cut-points for discriminating between sedentary, light, moderate, vigorous, very vigorous and extremely vigorous activity when assessing the physical activity intensity in adult recreational marathoners aged 30–45 years. Our secondary aim was to determine these cut-points taking into account the marathoners’ sex, since females display lower record values in marathon compared to males.

We hypothesized that wrist-worn GENEA accelerometer may present high capacity of classifying the intensity of physical activity in middle-aged recreational marathoners, independently of sex.

## Material and methods

### Sample set

All participants of the Valencia Fundación Trinidad Alfonso EDP 2016 Marathon received an invitation email to participate in the current study. Two informative seminars were organized in order to fully explain the study design (aims, protocol, hypothesis, etc.) to those individuals who accepted the invitation (N = 456). A total of 98 recreational marathon runners (83 males and 15 females) were selected to participate in this study, according to the following inclusion criteria: (1) age between 30 and 45 years; (2) body mass index (BMI) between 16 and 24.99 kg·m^-2^; (3) previous marathon experience, having a performance best time in marathon between 3 and 4 hours for males and 3:30 and 4:30 hours for females; and (4) healthy individuals who were free from cardiac or renal disease and from consuming drugs.

### Ethics statement

All individuals included in the current study were fully informed and gave their written consent to participate. The research was conducted according to the Declaration of Helsinki, and it was approved by the Research Ethics Committee of the Jaume I University of Castellon. This study is enrolled in the ClinicalTrails.gov database, with the code number NCT03155633 (www.clinicaltrials.gov).

### Data collection and analysis

A standardized questionnaire was used to collect demographic information as well as medical information, training plan and competition history (see S1 File for details).

Before performing the cardiopulmonary tests, anthropometric data of all the individuals were evaluated. Height was measured using a SECA 213 portable stadiometer (Seca GmbH & Co. Kg, Hamburg, Germany). Body mass was assessed with light sport clothing and barefoot using a Tanita MC-780 U (Tanita Corporation, Arlington Heights, IL). BMI was then calculated (height·mass^-2^). Bioelectrical impedance analyses (Tanita MC-780 U) was also used to determine body composition for all individuals, according to manufacturer’s protocol.

Each participant was then asked to complete a cardiopulmonary exercise test, which was done on a treadmill (pulsar^®^ 3p, h/p/cosmos sports & medical gmbh, Nussdorf-Traunstein, Germany) until exhaustion. Breath-by-breath gas exchange was measured by the Jaeger MasterScreen^®^ CPX gas analyzer. Gas analysis system was calibrated before each testing session. The run exercise test performed was an adaptation of the incremental ramp exercise protocol [37,38]. Initially, participants were standing on the treadmill for one minute. Then, speed was progressively increasing until reaching 6 km·h^-1^, where participants warmed-up for three minutes. After the warming-up period, speed was growing by 1 km·h^-1^ per minute from 8 km·h^-1^ to 11 or 12 km·h^-1^ when examining females or males, respectively. This speed was maintained during three minutes. Finally, speed was again increasing by 1 km·h^-1^ per minute until participant exhaustion. The breath-by-breath *V̇*O_2_ data collected by the gas analysis system was averaged per minute for further analysis. Arterial tension was measured each 3 min of the exercise test by using a Tango M2 Blood Pressure Monitor (GE Healthcare, Finland). Additionally, heart activity was evaluated throughout exercise test by using an electrocardiograph 1200W Digital RF Wireless System (Norav Medical, Germany).

During the course of the cardiopulmonary exercise test, participants wore a GENEActiv accelerometer (Activinsights Ltd., Kimbolton, Cambridgeshire, United Kingdom). The accelerometer was worn on the non-dominant wrist as a watch. Accelerometers were adjusted to record acceleration data at a rate of 85.7 Hz. Accelerometry data was collected at this frequency because of two different reasons: 1) to follow the same methodology than Esliger *et al*. (2011), and 2) to be able to collect information during 10 days (allowing us to monitor runners from 24h before to 9 days after the marathon).

Devices were calibrated by the manufacturer prior to use. Accelerometer devices were time synchronized with the gases analysis software. Acceleration data of each individual was downloaded using the GENEActiv software (Version 2.9). The BIN file created by the device was firstly converted to a CSV file. Then, the data was exported to a standard Excel file (Microsoft Excel 2013, Microsoft Corporation, Redmond, WA). We used the acceleration data to provide a Signal Magnitude Vector gravity-subtracted (SVMgs) per minute [25].

### Statistical analysis

To establish cut-points for the GENEA accelerometers, each minute of the run exercise test was then classified into one of the six relative-intensity categories: sedentary (<10% of *V̇*O_2max_), light (10 ≤ *X* < 25% of *V̇*O_2max_), moderate (25 ≤ *X* < 45% of *V̇*O_2max_), vigorous (45 ≤ *X* < 65% of *V̇*O_2max_), very vigorous (65 ≤ *X* < 85% of *V̇*O_2max_), and extremely vigorous (≥85% of *V̇*O_2max_) (Table 1). This classification was based on previous studies [16,17,23]. Then, the *V̇*O_2_ data per minute was converted to METs according to the standard conversion (1 MET = 3.5 ml·kg^-1^·min^-1^), with the aim to transform the breath-to-breath *V̇*O_2_ values to the energy consumption rate of physical activities [17,28]. Next, the METs per minute was recoded into binary indicator variables (0 or 1). Binary codification was based on the relative-intensity categories: sedentary (non-sedentary *versus* sedentary), light (less than light *versus* light to extremely vigorous), moderate (less than moderate *versus* moderate to extremely vigorous), vigorous (less than vigorous *versus* vigorous to extremely vigorous), very vigorous (less than very vigorous *versus* very to extremely vigorous), and extremely vigorous (less than extremely vigorous *versus* extremely vigorous).

**Table 1:** Relative-intensity categories of physical activity according to individualized V̇O_2max_ measured in 98 adult marathon runners.

Relative-intensity levels of physical activity ^#^	All samples (N = 98)		Males (N = 83)		Females (N = 15)	
	*V̇*O_2_ (ml·kg^-1^·min^-1^)	METs *	*V̇*O_2_ (ml·kg^-1^·min^-1^)	METs *	*V̇*O_2_ (ml·kg^-1^·min^-1^)	METs *
Sedentary *X* < 10%	*V̇*O_2_ < 5.45	METs < 1.56	*V̇*O_2_ < 5.57	METs < 1.59	*V̇*O_2_ < 4.82	METs < 1.38
Ligth 10% ≤ *X* <25%	5.45 ≤ *V̇*O_2_ <13.63	1.56 ≤ METs < 3.90	5.57 ≤ *V̇*O_2_ < 13.94	1.59 ≤ METs < 3.97	4.82 ≤ *V̇*O_2_ < 12.07	1.38 ≤ METs < 3.45
Moderate 25% ≤ *X* < 45%	13.63 ≤ *V̇*O_2_ < 24.54	3.9 ≤ METs < 7.01	13.94 ≤ *V̇*O_2_ < 25.08	3.97 ≤ METs < 7.15	12.07 ≤ *V̇*O_2_ < 21.72	3.45 ≤ METs < 6.21
Vigorous 45% ≤ *X* < 65%	24.54 ≤ *V̇*O_2_ < 35.44	7.01 ≤ METs < 10.13	25.08 ≤ *V̇*O_2_ < 36.23	7.15 ≤ METs < 10.33	21.72 ≤ *V̇*O_2_ < 31.38	6.21 ≤ METs < 8.97
Very Vigorous 65% ≤ *X* < 85%	35.44 ≤ *V̇*O_2_ < 46.35	10.13 ≤ METs < 13.24	36.23 ≤ *V̇*O_2_ < 47.38	10.33 ≤ METs < 13.50	31.38 ≤ *V̇*O_2_ < 41.03	8.97 ≤ METs < 11.72
Extremely Vigorous *X* ≥ 85%	*V̇*O_2_ ≥ 46.35	METs ≥ 13.24	*V̇*O_2_ ≥ 47.38	METs ≥ 13.50	*V̇*O_2_ ≥ 41.03	METs ≥ 11.72

Abbreviations: N, number of individuals; V̇O_2max_, maximum oxygen consumption; MET, metabolic equivalent task

Each minute of the cardiopulmonary test was classified into one of the six intensity categories of physical activity relative to an individual’s level of cardiorespiratory (*V̇*O_2max_).

* 1 MET = 3.5 ml·kg^-1^·min^-1^

^#^ *X* denotes the percentage of a person’s aerobic capacity (*V̇*O_2max_) used to classify each one of the six relative-intensity categories

The binary-coded MET data and SVM_gs_ per minute were exported to R software in order to accomplish a receiver operating characteristic (ROC) curve analysis. ROC analysis was adopted to evaluate the potential of using accelerometer data to distinguish between the different relative-intensity categories. The Youden Index method was used to set the optimal cut-point–the point on the curve at which (sensitivity + specificity − 1) is maximised. Therefore, the cut-point that optimizes the classification ability of accelerometry data, when equal weight is given to sensitivity and specificity, is established as the optimal cut-point [25,39]. Basic prediction accuracy parameters–including the area under the ROC curve (AUC), sensitivity and specificity–were calculated. ROC AUC values varies between 0 and 1, where 0.5 denotes a bad diagnostic test and 1 denotes an excellent diagnostic test. The ability of accelerometry data to distinguish between the different relative-intensity categories was inferred as follow: excellent (AUC = 0.90–1.00); good (AUC = 0.80–0.90); fair (AUC = 0.70–0.80); poor (AUC = 0.60–0.70); and fail (AUC = 0.50–0.60).

This analysis was carried out for each one of the six relative-intensity categories. Indeed, ROC analysis was done for all individuals, as well as for males and females separately. Note that ROC analysis were performed including data from 0 to 16 min for males, excluding data from 17 and 18 min since only 17 individuals were able to continue running after 16 min of the exercise test. For females, data from 0 to 15 min was used.

Finally, Spearman’s correlation test was used to known whether there was a linear correlation between SVMgs and METs. This test was used due to a non-normal distribution of the accelerometer data, according to Kolgomorov-Smirnov test. Statistical analysis was done using R software, and *p*-values lower than 0.05 were considered as statistically significant.

## Results

Detailed description of individuals regarding anthropometric data evaluated, as well as demographic information, medical information, training plan and competition history, is summarized in Table 2.

**Table 2:** Population description.

Variable		All sample (N = 98)	Males (N = 83)	Females (N = 15)
Physiological characteristics *	**age**	38.72 ± 3.63	38.76 ± 3.65	38.50 ± 3.63
	**BMI**	22.87 ± 1.71	23.18 ± 1.48	21.32 ± 2.01
	**% body fat**	14.74 ± 3.25	13.81 ± 3.67	19.54 ± 4.16
	**right-handed**	91	76	15
	**left-handed**	7	7	0
	***V̇*O**_**2max**_ **(ml·kg**^**-1**^**·min**^**-1**^**)**	54.53 ± 5.63	55.74 ± 5.14	48.27 ± 3.60
	**maximum METs**	15.54 ± 1.62	15.92 ± 1.46	13.72 ± 1.02
Training indicators*	**years of running**	6.49 ± 2.81	6.58 ± 2.91	5.38 ± 1.80
	**sessions per week**	4.81 ± 0.86	4.90 ± 0.85	4.33 ± 0.81
	**kilometers per week**	63.16 ± 13.42	64.45 ± 13.21	55.66 ± 12.79
	**hours per week**	7.30 ± 2.67	7.46 ± 2.69	6.21 ± 2.27
History as marathoner *	**marathons finished**	3.28 ± 3.00	3.56 ± 3.09	1.92 ± 2.08
	**marathon per year**	1.09 ± 0.61	1.21 ± 0.61	0.93 ± 0.59
Work intensity ^#^	**high intensity**	7.07%	8.43%	0%
	**medium intensity**	31.31%	31.32%	31.25%
	**low intensity**	61.61%	60.24%	68.75%
Levels of study ^#^	**school graduate**	5.10%	4.87%	6.25%
	**high school graduate**	6.12%	6.09%	6.25%
	**professional certificate**	16.32%	18.29%	6.25%
	**undergraduate degree**	72.4%	70.73%	81.25%

Abbreviations: N, number of samples; BMI, body mass index; SD, standard deviation

* Values are presented as mean ± SD

^#^ Values are presented as percentage of all individuals, males and females

A total of 98 participants (83 males and 15 females) completed a cardiopulmonary exercise test until exhaustion. Table 3 recapitulates the results of the cardiopulmonary exercise tests per minute for all individuals, as well as for males and females separately. Exercise time length was higher in males than in females. All males completed 12 min running on the treadmill, and progressively interrupted their exercise test due to fatigue after that minute. The best four males completed a total of 18 min, being the treadmill velocity of 19 km·h^-1^. Among female participants, exercise test duration was not less than 12 min. Two out of 15 females were able to complete a total of 15 min running, being the treadmill velocity of 16 km·h^-1^. Each minute of the run exercise test was then classified into one of the six relative-intensity categories by taking into account a person’s aerobic capacity (*V̇*O_2max_). The intensity of each relative-physical activity in METs and *V̇*O_2_ (ml·kg^-1^·min^-1^) is summarized in the Table 1.

**Table 3:** Mean values of energy expenditure (METs) and accelerometer output (SVM_gs_) for each minute of the 98 cardiopulmonary exercise tests.

Time (min)	Speed (km·h^-1^)	All individuals (N = 98)			Males (N = 83)			Females (N = 15)		
		N	METs *	SVM_gs_ (g·min)	N	METs *	SVM_gs_ (g·min)	N	METs *	SVM_gs_ (g·min)
0	0	98	1.46 ± 0.57	93.47 ± 65.82	83	1.46 ± 0.55	87.05 ± 57.75	15	1.49 ± 0.70	129.02 ± 94.28
1	0.5	98	1.44 ± 0.49	187.54 ± 155.86	83	1.48 ± 0.49	190.41 ± 164.10	15	1.21 ± 0.43	171.67 ± 101.39
2	6	98	2.73 ± 0.72	857.32 ± 462.82	83	2.76 ± 0.76	870.25 ± 483.22	15	2.54 ± 0.38	785.79 ± 331.65
3	6	98	4.70 ± 0.67	1405.01 ± 424.64	83	4.68 ± 0.70	1381.87 ± 387.93	15	4.82 ± 0.46	1533.02 ± 589.21
4	6	98	5.30 ± 0.61	1624.80 ± 584.02	83	5.29 ± 0.64	1595.57 ± 608.89	15	5.36 ± 0.41	1786.54 ± 398.27
5	8	98	6.40 ± 0.89	3269.14 ± 1009.90	83	6.39 ± 0.95	3281.01 ± 1028.88	15	6.48 ± 0.42	3203.45 ± 927.77
6	9	98	8.66 ± 0.73	4455.89 ± 862.04	83	8.67 ± 0.76	4511.70 ± 816.02	15	8.61 ± 0.55	4147.08 ± 1061.97
7	10	98	9.58 ± 0.74	4837.47 ± 986.13	83	9.60 ± 0.76	4846.79 ± 972.38	15	9.46 ± 0.61	4785.89 ± 1093.78
8	11	98	10.32 ± 0.75	5145.54 ± 1101.79	83	10.34 ± 0.78	5132.55 ± 1086.95	15	10.23 ± 0.62	5217.48 ± 1218.38
9	12 / 11 ^#^	98	11.05 ± 0.99	5497.10 ± 1192.59	83	11.08 ± 1.04	5489.30 ± 1197.44	15	10.93 ± 0.65	5540.25 ± 1205.68
10	12 / 11 ^#^	98	11.68 ± 0.82	5716.85 ± 1260.17	83	11.81 ± 0.77	5725.23 ± 1275.23	15	10.96 ± 0.78	5670.45 ± 1213.19
11	12	98	12.01 ± 0.82	5826.97 ± 1289.95	83	12.13 ± 0.80	5776.95 ± 1291.03	15	11.36 ± 0.66	6103.74 ± 1292.11
12	13	98	12.29 ± 0.85	6144.29 ± 1410.26	83	12.37 ± 0.82	6048.91 ± 1393.39	15	11.80 ± 0.84	6672.02 ± 1433.74
13	14	96	12.90 ± 0.88	6746.56 ± 1661.72	83	12.97 ± 0.87	6617.35 ± 1577.41	13	12.47 ± 0.86	7571.53 ± 2001.21
14	15	90	13.64 ± 0.89	7337.31 ± 1714.91	81	13.67 ± 0.90	7228.05 ± 1648.30	9	13.36 ± 0.74	8320.66 ± 2083.25
15	16	76	14.36 ± 0.94	8027.57 ± 2046.65	74	14.38 ± 0.95	8056.41 ± 1799.32	2	13.58 ± 0.12	10974.31 ± 370.23
16	17	52	15.13 ± 1.02	9101.20 ± 2038.07	52	15.13 ± 1.02	9101.20 ± 2038.07	-	-	-
17	18	17	16.29 ± 1.02	9780.52 ± 2683.43	17	16.29 ± 1.02	9780.52 ± 2683.43	-	-	-
18	19	4	16.72 ± 0.97	11745.28 ± 3470.36	4	16.72 ± 0.97	11745.28 ± 3470.36	-	-	-

Abbreviations: N, number of samples; MET, metabolic equivalent task; SVM_gs_, signal magnitude vector gravity-subtracted; SD, standard deviation

Values are presented as mean ± SD

* 1 MET = 3.5 ml·kg^-1^·min^-1^

^#^ Treadmill speed at 12 or 11 km·h^-1^ when examining males or females, respectively

Table 4 summarizes the results of the ROC curve analyses performed to establish cut-points for the GENEA devices. Cut-points in SVM_gs_, sensitivity and specificity values, and the area under the curve (AUC) were estimated for all six relative-intensity categories of physical activity. ROC analyses revealed that GENEA devices were able to distinguish between all relative-intensity levels, presenting AUC values ranging from 0.881 to 0.995. Indeed, sensitivity and specificity values were reasonably high, confirming the great overall capability to discriminate between sedentary, light, moderate, vigorous, very vigorous and extremely vigorous intensity levels of the wrist-worn GENEA devises. Regarding all different intensities, extremely vigorous category showed the lower AUC values (0.886 for all individuals, 0.881 for males and 0.924 for females), being the hardest intensity level to discriminate. Note that the reduced specificity and sensitivity for extremely vigorous intensity influenced the accurate classification of this relative-intensity category (Table 4).

**Table 4:** Performance analysis of wrist-worn GENEA cut-points for each intensity level in adult marathon runners.

Intensity level of physical activity	Sensitivity (%)	Specificity (%)	Area under the ROC curve (95% CI)	Youden Index	GENEA cut-points in SVM_gs_ (g∙min) *
**All samples (N = 98)**					
Sedentary	99.2	93.6	0.973 (0.966–0.980)	0.928	SVM_gs_ < 528.31
Light	93.6	99.2	0.973 (0.966–0.980)	0.928	528.31 ≤ SVM_gs_ < 1166.28
Moderate	97.2	93.5	0.993 (0.990–0.996)	0.907	1166.28 ≤ SVM_gs_ < 3679.91
Vigorous	96.5	93.9	0.988 (0.984–0.993)	0.904	3679.91 ≤ SVM_gs_ < 4155.94
Very Vigorous	95.1	78.0	0.943 (0.933–0.954)	0.731	4155.94 ≤ SVM_gs_ < 5250.68
Extremely Vigorous	88.9	71.0	0.886 (0.867–0.905)	0.599	SVM_gs_ ≥ 5250.68
**Males (N = 83)**					
Sedentary	99.1	94.0	0.973 (0.966–0.981)	0.931	SVM_gs_ < 528.31
Light	94.0	99.1	0.973 (0.966–0.981)	0.931	528.31 ≤ SVM_gs_ < 1166.28
Moderate	97.0	93.2	0.992 (0.989–0.996)	0.902	1166.28 ≤ SVM_gs_ < 3679.91
Vigorous	97.6	93.8	0.99 (0.985–0.995)	0.914	3679.91 ≤ SVM_gs_ < 4364.64
Very Vigorous	91.7	80.9	0.94 (0.929–0.952)	0.726	4364.64 ≤ SVM_gs_ < 5264.37
Extremely Vigorous	89.9	70.3	0.881 (0.859–0.903)	0.602	SVM_gs_ ≥5264.37
**Females (N = 15)**					
Sedentary	100	93.0	0.968 (0.946–0.990)	0.930	SVM_gs_ < 326.08
Light	93.0	100	0.968 (0.946–0.990)	0.930	326.08 ≤ SVM_gs_ < 1264.59
Moderate	98.3	97.8	0.995 (0,989–1.000)	0.961	1264.59 ≤ SVM_gs_ < 2717.5
Vigorous	97.8	93.8	0.988 (0.977–0.999)	0.916	2717.5 ≤ SVM_gs_ < 3355.56
Very Vigorous	98.3	86.5	0.97 (0.951–0.989)	0.848	3355.56 ≤ SVM_gs_ < 5796.21
Extremely Vigorous	86.1	82.5	0.924 (0.883–0.965)	0.686	SVM_gs_ ≥ 5796.21

Abbreviations: N, number of samples; ROC, receiver operation curve; CI, coefficient interval; SVM_gs_, signal magnitude vector gravity-subtracted

* Optimal cut-points maximising Youden Index

**Fig 1:**
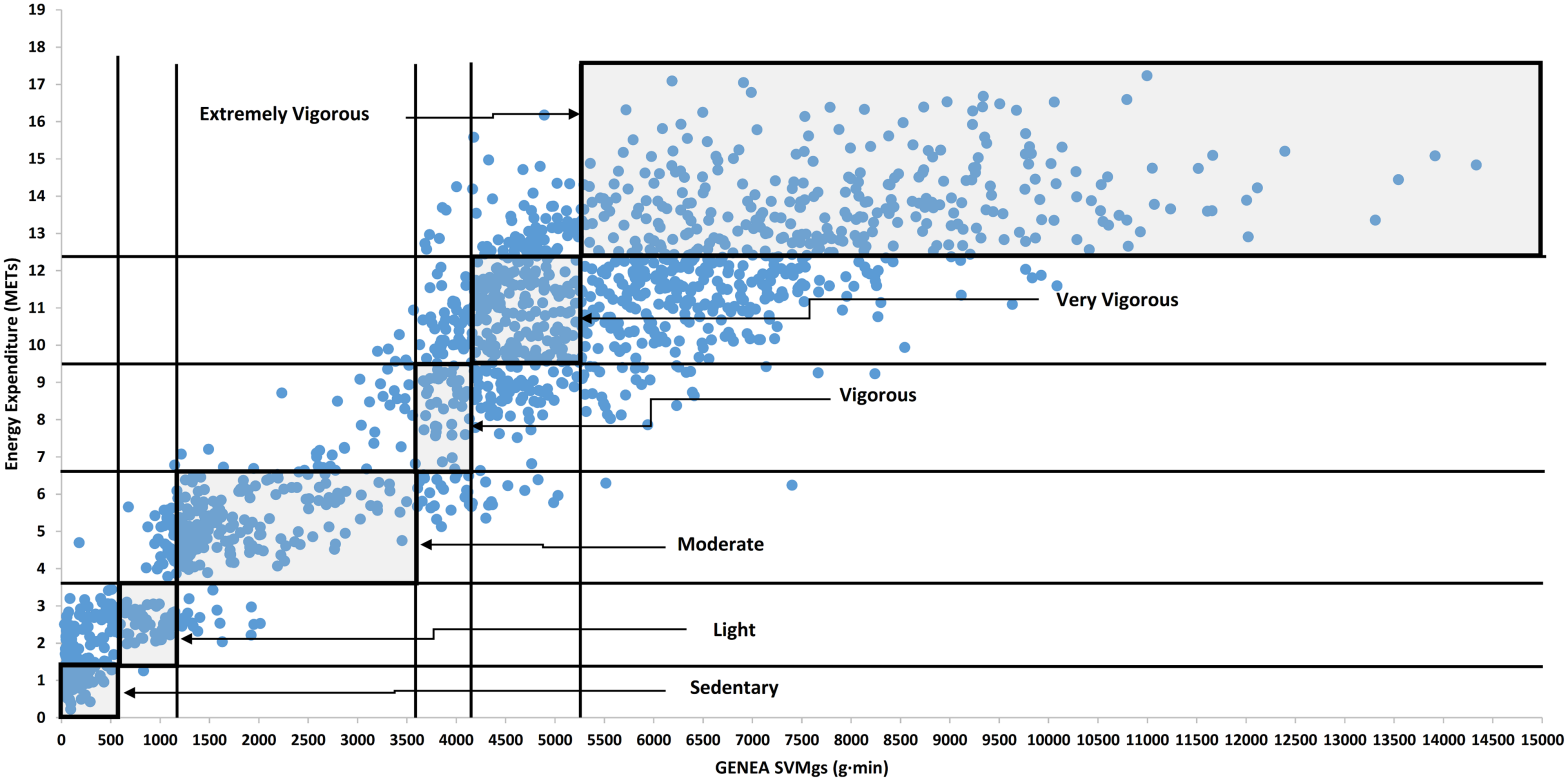
Correlation between the wrist-worn GENEA SVM_gs_ (g·min) and the energy expenditure (METs) along the 98 cardiopulmonary exercise tests. Vertical lines delimited the different relative-intensity levels according to SVM_gs_ cut-points estimated, and horizontal lines delimited the different relative-intensity levels according to METs cut-points measured (equivalent to *V̇*O_2max_ classification). Grey regions delimit the consensus outcome between the measured and predicted intensity categories, and all observations inside these regions are correct classifications for each intensity level. SVMgs, signal magnitude vector gravity-subtracted. MET, metabolic equivalent task.

Fig 1 illustrates the relationship between METs and SVM_gs_ for all individuals. Vertical lines delimited the different relative-intensity levels according to cut-points in SVM_gs_ estimated, and horizontal lines delimited the different relative-intensity levels according to cut-points in METs measured (equivalent to *V̇*O_2max_ classification). Therefore, grey regions delimit the consensus outcome between the measured and predicted intensity categories, and all observations inside these regions are correct classifications for each intensity level. The Spearman’s correlation test showed a high linear relationship between METs and SVM_gs_ when all individuals were analyzed together (rs = 0.886, *p*-value = 2.20x10^-16^), as well as when sample set was separated by sex (rs = 0.884 and *p*-value = 2.20x10^-16^ for males, rs = 0.912 and *p*-value = 2.20x10^-16^ for females).

## Discussion

The delineation and validation of intensity levels of physical activity from accelerometer data has been deeply studied in the last few years [17,20,21,40]. The GENEA accelerometer has been proposed as one of the most accurate tools to assess physical activity (in terms of intensity, frequency and duration) during free-living conditions. However, to our knowledge, this is the first time that researching have been focused on distinguish each relative-intensity activity level in adult recreational marathoners from accelerometer data. It is note that relative-intensity activities, rather than standard-intensity activities established for adult population [25], were used in this study since marathon runners present previous exercise experience and therefore higher relative level of fitness than standard adult population. Processing original accelerometer data to distinguish relative-intensity activity levels might provide valuable information for athletes, coaches and healthcare specialists, such us energy expenditures during daily activities, training sessions or over the course of a long-distance race.

Previous studies recommend being cautious using the GENEA cut-points when testing different populations and/or activities other than those on which the cut-points were specifically established [24,26,32]. For that reason, the main aim of the current study was to determine relative-intensity activity cut-points in middle-aged recreational marathoners using the GENEA accelerometer. This was done for six relative-intensity activity levels (sedentary, light, moderate, vigorous, very vigorous and extremely vigorous), which were established based on individualized *V̇*O_2max_. A total of 98 participants were collected for this primary purpose, being a significantly larger sample set compared to previous studies [22,24,25,29].

In this study, cardiopulmonary exercise test approach was performed with the individual running on a treadmill, rather than riding on a stationary bicycle, since individuals were marathon runners. The biomechanical differences between running and riding might influence the accelerometer data collection [26,40]. According to that, the accelerometer device was placed on the non-dominant wrist in order to record arm movement during running, as recommended by previous studies [17,21,26,36]. Body location of GENEA devices has been identified as an essential detail to take into account in physical activity monitoring studies [17,22,26,27,40].

Cut-points for the GENEA devices were established to optimize the balance between sensitivity and specificity (maximizing the Youden index), in order to guarantee the optimality of the cut-points. As expected, cut-points in SVM_gs_ were greater for sedentary, light, moderate and vigorous activity than these reported by Esligher *et al*. (2011). Besides marathon runners display greater level of fitness compared to normal population, these discrepancies might also be due to testing approach differences. In this study, we monitored runners during a continuous activity that progressively increases its intensity. However, Esligher *et al*. (2011) monitored adults performing a wide range of structured activities in a lab-based environment, classifying each activity as sedentary, light, moderate or vigorous activity. Indeed, Esligher *et al*. (2011) had a relatively small sample size (18 individuals for slow treadmill run, 14 for medium treadmill run, and 5 for fast treadmill run), which may limit their results for classifying vigorous activity.

Overall, the SVM_gs_ cut-points established in this study were able to efficiently classify different activities with a good to excellent accuracy. Since no previous studies have used a similar methodology as well as equivalent sample population, we are not able to perform a comprehensive comparison of our classification accuracy values. Our results revealed a classification accuracy of 94.08% for sedentary, 94.08% for light, 96.54% for moderate, 95.53% for vigorous, 86.27% for very vigorous, and 73.61% for extremely vigorous activity. An important advance of the current study is the ability to split vigorous activities into three different intensity categories: vigorous, very vigorous and extremely vigorous. Enlarging the range of physical activity intensities covered by adding new activity categories allow to accurately calculate energy expenditure for activities with higher than 7 METs values (i.e. running values commonly range from 8 to 18 METs [28,41]).

In this regard, our correlation analyses reported that the GENEA devices explained 78.50% of the total variance in energy expenditure (rs^2^ = 0.785), suggesting that the triaxial accelerometers (worn on the non-dominant wrist) can be used to predict energy expenditure for running activities with high metabolic cost (≥7 METs). However, the estimation accuracy of energy expenditure in METs from accelerometer data was slightly reduced at extremely vigorous activity because fatigue has been revealed to interfere in running biomechanics, as shown by natural arm and legs movement alteration [42], increasing therefore the standard deviation of SVM_gs_ collected by the accelerometer device. Besides, the number of data points collected at extremely vigorous activity was reduced–runners were progressively stopped because of exhaustion.

Linear correlation between SVM_gs_ and METs reported by previous studies for wrist-worn accelerometers was slightly lower than our correlation values [25,26]. Reasonably, the homogeneity of the sample set (adult recreational marathoners with similar age, body mass index, and level of fitness) is the reason of having a remarkably correlation between SVM_gs_ and METs. Because of the main purpose of this study was to establish cut-points in adult recreational marathoners, we carefully selected individuals that represents this specific population subset. For example, individuals aged between 30 and 45 years were selected because it is the age group with higher number of marathon participants [1,2,43]. Indeed, their performance in terms of running speed appeared to be unaffected by their age [1–3,43]. Consequently, our relative-intensity activity cut-points are not applicable for adult marathon runners older than 45 years, being necessary to estimate specific cut-points in SVM_gs_ for other age groups. Therefore, it is recommended to establish specific cut-points for a specific population subset in order to accurately predict energy expenditure by using accelerometer devices.

It is well-known that there are essential physical differences between males and females with regard to sport performance [2,5,16,44]. Accordingly, we performed all cut-point analysis separating the sample set by sex. Males showed higher *V̇*O_2max_, and therefore higher MET cut-points for each relative-intensity activities, compared to females (see Table 1). In general, the ability of GENEA devices for classifying activity intensities was relatively greater in females than in males. However, given the small number of female participants (N = 15), results obtained should be cross-validated in a largest population. The reason for having small number of females is that only a 14.20% of finishers in the Valencia Fundación Trinidad Alfonso EDP 2016 Marathon were females. In this study, the percentage of females was 15.15%. To confirm our results, future research determining the SVM_gs_ cut-points should be achieved in a larger population of female marathon runners aged from 30 to 45 years.

Several strengths and limitations are noteworthy in the present study. To our knowledge, this is the first study focused on distinguish between different intensities of physical activity levels in middle-aged recreational marathoners from accelerometer data. The well-controlled experimental design allowed us to delineate specific GENEA cut-points for a robust assessment of physical activity intensity level. Finally, the homogeny and large population used was essential for ensuring the optimality of the cut-points. The main limitation of this study was that measures were not performed in free-living conditions.

Since the present study was lab-based, future validation of the SVM_gs_ cut-points in an independent sample set of adult recreational marathoners running in free-living conditions for optimal practical applications. Cross-validation would be assist in quantifying energy expenditure during the course of a marathon race. Besides, monitoring runners during non-training activities would allow comparing sedentary and light cut-points in this specific population with these previously established for standard population.

In conclusion, the GENEA accelerometer have been able to efficiently classify between all six-relative intensity levels of physical activity in adult recreational marathoners aged between 30 and 45 years. The GENEA accelerometer presents an excellent intensity classification accuracy when applying GENEA cut-points established for all samples, males and females. Remarkably, correlation tests showed a high linear relationship between energy expenditure (expressed as METs) and GENEA estimated SVM_gs_ when all individuals were analyzed together, as well as when sample set was separated by sex. Therefore, the GENEA accelerometer could be a useful tool for athletes, coaches and healthcare specialists to measure energy expenditure during races and training sessions, but also to monitor daily routine activities and rest time.

## Supporting information

S1 File Standardized questionnaire from data collection. Questionnaire used to collect information from participants in Spanish and English.
(PDF)

S2 File Raw data of the study. (XLSX)
