# Supplementary material for: Establishing cut-points for physical activity classification using triaxial accelerometer in middle-aged recreational marathoners
Source: PLoS One. 2018 Aug 29;13(8):e0202815. doi: 10.1371/journal.pone.0202815 (PMC6114871; doi:10.1371/journal.pone.0202815)
Supplement: S1 File — Questionnaire used to collect information from participants in Spanish and English. (PDF) [file pone.0202815.s001.pdf]

CUESTIONARIO ESTANDARIZADO

A) BLOQUE I: DATOS PERSONALES

1- Nombre y Apellidos

2- DNI

3- Estado Civil

- Separado/a-Divorciado/a-Soltero/a
- Casado/a-En pareja

4- Nº de Hijos/as

- Ninguno
- 1 hijo/a
- 2 hijos/as
- Más de 2 hijos/as

5- Nivel de Estudios

- Ninguno
- Graduado Escolar
- Bachillerato
- Formación Profesional
- Estudios Universitarios

6- Ocupación Laboral

- Con alta intensidad de ejercicio físico
- Con media intensidad de ejercicio físico
- Con baja intensidad de ejercicio físico

7- Horario Laboral

- Fijo
- Rotativo(Turnos)

8- En caso de tener un horario laboral fijo, ¿Puedes especificar franja horaria?)

- Mañanas
- Tardes
- Jornadas Partidas

9- En caso de tener un horario de Jornada Partida, por favor indícanos tu horario de trabajo.

10- ¿Eres trabajador/ra del Grupo de Hospitales NISA?

- Si
- No

B) BLOQUE II: HÁBITOS GENERALES DEL ENTRENAMIENTO

1- ¿Tienes entrenador en la actualidad?
  - Sí
  - No

2- En caso afirmativo, ¿Cuántos años hace que lo tienes?
  - Menos de 1 año
  - 1 año
  - 2 años
  - 3 años
  - Más de 3 años

3- ¿Tienes nutricionista en la actualidad?
  - Sí
  - No

4- En caso afirmativo, ¿Cuantos años hace que lo tienes?
  - Menos de 1 año
  - 1 año
  - 2 años
  - 3 años
  - Más de 3 años

5- ¿Tomas complementos nutricionales en la preparación para el maratón?
  - Sí
  - No

6- En caso afirmativo, ¿Qué productos tomas?

7- ¿Tomas complementos nutricionales para la recuperación del maratón?
  - Sí
  - No

8- En caso afirmativo, ¿Qué productos tomas?

9- ¿Te haces masajes habitualmente?
  - Sí
  - No

10- En caso afirmativo, ¿Cuántos masajes te realizas aproximadamente en un mes?
  - 1
  - 2
  - 3
  - 4

- 5
- 6
- 7
- 8
- 9
- 10
- Más de 10

11- ¿Cuántos años llevas corriendo de forma habitual?

- Menos de 1 año
- 1
- 2
- 3
- 4
- 5
- 6
- 7
- 8
- 9
- 10
- Más de 10

12- ¿Cuántos maratones de asfalto has corrido? (Sólo número)

13- ¿Cuántos maratones de asfalto has finalizado? (Sólo número)

14- ¿Cuántos maratones de asfalto corres al año? (Sólo número)

15- Habitualmente, después de un maratón, ¿Cuántos días dejas de recuperación para volver a correr?

- 1
- 2
- 3
- 4
- 5
- 6
- 7
- Más de 7

16- ¿Has sufrido alguna lesión desde el 1 de septiembre de 2015?

- Sí
- No

17- En caso afirmativo, ¿Puedes indicarnos de qué tipo?

- Lesiones musculares (Roturas fibrilares, Contracturas, etc.)
- Lesiones óseas (Fracturas de estrés, Fisuras, etc.)
- Lesiones tendinosas (Tendinitis, roturas de tendón, etc.)

18- ¿Cuál es la cantidad aproximada de líquido que acostumbras a beber en una carrera de Maratón?

- Menos de medio litro
- Entre medio litro y un litro
- Entre litro y un litro y medio
- Entre un litro y medio y dos litros
- Más de dos litros

19- ¿Cuánto tiempo suele transcurrir habitualmente entre tu último entrenamiento (Más de 5 km de carrera) y un maratón?

- 24 horas
- 48 horas
- 72 horas
- 5 días
- 7 días o más

C) BLOQUE III: PLANIFICACIÓN DEL ENTRENAMIENTO

1- ¿Cuántos días a la semana entrenas?

- 1
- 2
- 3
- 4
- 5
- 6
- 7

2- De los días de entrenamiento que realizas a la semana, ¿Cuántos son en doble sesión de entrenamiento?

- Ninguno
- 1

- 2
- 3
- 4
- 5
- 6
- 7

3- ¿Cuántos kilómetros realizas aproximadamente en una semana tipo de entrenamiento? (Sólo número)

4- ¿Cuántas horas realizas aproximadamente en una semana tipo de entrenamiento? (Sólo número)

5- ¿Cuál es la superficie por la que sueles entrenar y/o correr habitualmente? *

- Asfalto / hormigón
- Hierba
- Tierra
- Playa
- Montaña
- Otra

6- ¿Cuál es tu marca personal en 10k homologada? (Respuesta en formato: hh:mm:ss)

7- ¿Cuál es tu marca personal en Media Maratón homologada? (Respuesta en formato: hh:mm:ss)

8- ¿Cuál es tu marca objetivo para el Maratón de Valencia Fundación Trinidad Alfonso EDP 2016? (Tipo de respuesta en formato: hh:mm:ss)

9- ¿El Maratón de Valencia Fundación Trinidad Alfonso EDP 2016 es tu objetivo principal de la temporada?

- Si
- No

10- ¿Cuándo empezaste a entrenar específicamente para el Maratón de Valencia Fundación Trinidad Alfonso EDP 2016? (Respuesta en formato: mes y año)

11- ¿Vas a correr alguna media maratón para preparar el Maratón de Valencia Fundación Trinidad Alfonso EDP 2016?

- Si
- No

12- En caso afirmativo, ¿Cuántas?

- 1
- 2
- 3

- Más de 3

D) BLOQUE IV: METODOLOGIA DEL ENTRENAMIENTO

1- ¿Utilizas el gimnasio en la planificación de tu entrenamiento? *

- Sí
- No

2- En caso afirmativo, ¿Cuántas sesiones realizas de gimnasio?

- 1 al mes
- 1 cada quince días
- 1 a la semana
- 2 a la semana
- 3 a la semana
- Más de 3 a la semana

3- ¿Incluyes sesiones de natación en la planificación de tu entrenamiento? *

- Sí
- No

4- En caso afirmativo, ¿Cuántas sesiones realizas de piscina?

- 1 al mes
- 1 cada quince días
- 1 a la semana
- 2 a la semana
- 3 a la semana
- Más de 3 a la semana

5- ¿Incluyes sesiones de ciclismo en la planificación de tu entrenamiento? *

- Sí
- No

6- En caso afirmativo, ¿Cuántas sesiones realizas de ciclismo?

- 1 al mes
- 1 cada quince días
- 1 a la semana
- 2 a la semana
- 3 a la semana
- Más de 3 a la semana

BLOQUE V: PARÁMETROS DE SALUD

1- ¿Eres Fumador?
   - Sí
   - No
2- En caso afirmativo, ¿Cuantos cigarrillos al día?
   - Menos de 5
   - Entre 5 y 10
   - Entre 10 y 20
   - Más de 20
3- ¿En alguna ocasión te han diagnosticado hipertensión?
   - Sí
   - No
4- ¿Toma algún medicamento antihipertensivo?
   - Si
   - No
5- En caso afirmativo, ¿Puedes indicarnos el nombre del medicamento?
6- ¿En alguna ocasión te han diagnosticado de diabetes?
   - Si
   - No
7- En caso afirmativo, ¿Puedes indicarnos el tratamiento que empleas? (Responde a cada una con un SI/NO/LO DESCONOZCO)
   - Sólo dieta y ejercicio
   - Pastillas antidiabéticas orales
   - Insulina inyectada
   - Bomba subcutánea de infusión continua de insulina
8- ¿En alguna ocasión te han diagnosticado de alguna enfermedad autoinmune (lupus eritematoso, artritis reumatoide, crioglobulinemia, vasculitis)? *
   - Sí
   - No
9- ¿En alguna ocasión te han diagnosticado de alguna enfermedad renal, o has precisado valoración por un Nefrólogo? *
   - Sí
   - No
10- En caso afirmativo, ¿Puedes indicarnos el diagnóstico si lo conoces? (Responde a cada una con un SI/NO/LO DESCONOZCO)
   - Hemataurina

- Glomerulonefritis
- Cólicos nefríticos o litiasis renales
- Enfermedad Vascular renal. (Estenosis de arteria renal, trombosis de vena renal)
- Riñón único
- Nefropatía diabética
- Afección renal por enfermedad sistémica (Lupus, Vasculitis)

11- ¿En alguna ocasión has precisado atención por problemas urológicos (Ejemplo cólicos nefríticos, problemas en la vejiga o en la próstata)?

- Sí
- No

12- En caso afirmativo, ¿Puedes indicarnos el diagnóstico si lo conoces? (Responde a cada una con un SI/NO/LO DESCONOZCO)

- Cólico Renoureteral
- Lesión intravesical
- Malformación ureteral ó uretral.
- Problemas de próstata.

13- ¿En alguna ocasión has observado sangre en la orina después de un esfuerzo físico muy intenso?

- Sí
- No

14- ¿En alguna ocasión has observado espuma en la orina después de un esfuerzo físico muy intenso?

- Sí
- No

15- ¿Consumes habitualmente fármacos analgésicos de tipo antiinflamatorio? (Ejemplo: ibuprofeno, espidifen, diclofenaco, dexketoprofeno, voltaren, enantyum, aspirina, ácido acetilsalicílico, airtal)

- Sí
- No

STANDARIZED QUESTIONNARIE

A) PART I: PERSONAL DATA

2- Name and Surnames

3- NID

4- Civil Status

- Separated – Divorced - Single
- Married – In couple

5- Number of children

- None
- 1
- 2
- More than 2

6- Study level

- None
- School graduate
- High school graduate
- Professional certificate
- Undergraduate degree

7- Professional occupations

- With high intensity of physical exercise
- With medium intensity of physical exercise
- With low intensity of physical exercise

8- Working schedule

- Fixed
- Rotating (Shift schedule)

9- If you have a fixed work schedule, could you specified it?

- Continuous shift in the mornings
- Continuous shift in the afternoon/evening
- Split shift

10- If you have a split work Schedule, please indicate your work schedule.

11- Are you worker of NISA Hospitals Group?

- Yes
- No

B) PART II: GENERAL TRAINING HABITS

1- Nowadays, do you have coach?
   - Yes
   - No
2- If yes, how many years have you working with him/her?
   - Less than 1 year
   - 1 year
   - 2 years
   - 3 years
   - More than 3 years
3- Nowadays, do you have nutritionist?
   - Yes
   - No
4- If yes, how many years have you working with him/her?
   - Less than 1 year
   - 1 year
   - 2 years
   - 3 years
   - More than 3 years
5- Do you take nutritional supplements for preparing the marathon?
   - Yes
   - No
6- If yes, which ones?
7- Do you take nutritional supplements for recovering after the marathon?
   - Yes
   - No
8- If yes, which ones?
9- Do you usually get a massage?
   - Yes
   - No
10- If yes, how many massages do you get per month?
   - 1
   - 2
   - 3
   - 4

- 5
- 6
- 7
- 8
- 9
- 10
- More than 10

12- How many years have you regularly been running?

- Less than one year
- 1
- 2
- 3
- 4
- 5
- 6
- 7
- 8
- 9
- 10
- More than 10 years

13- How many road marathons has you done? (Give a number)

14- How many road marathons has you finished? (Give a number)

15- How many road marathons do you run per year? (Give a number)

16- After running a marathon, how many days do you normally rest before starting to run again?

- 1
- 2
- 3
- 4
- 5
- 6
- 7
- More than 7

17- Have you suffered any injury since September 1, 2015?
  - Yes
  - No
18- If yes, could you specify the type of your injury?
  - Muscular injuries (Fibrillar rupture, muscle contractures, etc.)
  - Bone injuries (Stress fracture, bone fissures, etc.)
  - Tendinous injuries (Tendinitis, tendon ruptures, etc.)
19- Approximately, what is the amount of liquid that you usually drink during a marathon race?
  - Less than 1 litre
  - Between 0.5 and 1 litre
  - Between 1 and 1.5 litres
  - Between 1.5 and 2 litres
  - More than 2 litres
20- How long does it usually take between your last training (more than 5 km of running) and a marathon?
  - 24 h
  - 48 h
  - 72 h
  - 5 days
  - 7 days or more

C) PART III: TRAINING PROGRAMATION

1- How many days do you train per week?
  - 1
  - 2
  - 3
  - 4
  - 5
  - 6
  - 7
2- Out of all the training days you do per week, how many do you perform a double session?
  - None
  - 1

- 2
- 3
- 4
- 5
- 6
- 7

3- How many kilometers do you approximately perform per week? (Give a number)

4- How many hours do you approximately perform per week? (Give a number)

5- What is the surface where do you usually train/run?

- Asphalt/Concrete
- Grass
- Ground
- Beach
- Mountain
- Others

6- What is your homologated personal best in 10k? (Answer in format: hours:minutes:seconds)

7- What is your homologated personal best in half marathon? (Answer in format: hours:minutes:seconds)

8- What is your target time for the Valencia Fundación Trinidad Alfonso EDP marathon? (Answer in format: hours:minutes:seconds)

9- Is the Valencia Fundación Trinidad Alfonso EDP marathon your main objective of the seasson?

- Yes
- No

10- When do you specifically start to train for the Valencia Fundación Trinidad Alfonso EDP marathon? (Answer in format: month and year)

11- Are you going to run a half marathon for preparing the Valencia Fundación Trinidad Alfonso EDP marathon?

- Yes
- No

12- If yes, how many?

- 1
- 2
- 3
- More than 3

D) PART IV: TRAINING METHODOLOGY

1- Do you include gym sessions in your training program?
- Yes
- No

2- If yes, how many sessions do you perfom?
- 1 per month
- 1 per 15 days
- 1 per week
- 2 per week
- 3 per week
- More than 3 per week

3- Do you include swim sessions in your training plan?
- Yes
- No

4- If yes, how many sessions do you perfom?
- 1 per month
- 1 per 15 days
- 1 per week
- 2 per week
- 3 per week
- More than 3 per week

5- Do you include bike sessions in your training plan?
- Yes
- No

6- If yes, how many sessions do you perfom?
- 1 per month
- 1 per 15 days
- 1 per week
- 2 per week
- 3 per week
- More than 3 per week

PART V: HEALTH PARAMETERS

1- Are you smoker?
  - Yes
  - No
2- If yes, how many cigarettes do you smoke per day?
  - Less than 5
  - Between 5 and 10
  - Between 10 and 20
  - More than 20
3- Have you ever been diagnosed with hypertension?
  - Yes
  - No

4- Do you take any antihypertensive medication?
  - Yes
  - No
5- If yes, which one?
6- Have you ever been diagnosed with diabetes?
  - Yes
  - No
7- If yes, could you specify which treatment do you use? (Answer each point writing Yes/No/I don't know)
  - Diet and exercise
  - Oral antidiabetic pills
  - Injected insulin
  - Subcutaneous insulin continuous infusion pump
8- Have you ever been diagnosed with any autoimmune disease (lupus erythematosus, rheumatoid arthritis, cryoglobulinemia, vasculitis)?
  - Sí
  - No
9- Have you ever been diagnosed with any renal disease, or have you ever required assessment by a nephrologist?
  - Yes
  - No

10- If yes, could you specify the diagnostic? (Answer each point writing Yes/No/I don't know)

- Hematuria
- Glomerulonephritis
- Nephritic colic or kidney stones
- Renal vascular disease (Renal artery stenosis, renal vein thrombosis)
- Single kidney
- Diabetic nephropathy
- Renal disease due to systemic disease (Lupus, Vasculitis)

11- Have you ever needed attention due to urological problems (renal colic, urinary problems in the bladder or prostate)?

- Yes
- No

12- If yes, could you specify the diagnostic? (Answer each point writing Yes/No/I don't know)

- Renal colic
- Intravesical injury
- Urethral and urethra malformation
- Prostate problems

13- Have you ever seen blood in your urine after performing a high intensity physical effort?

- Yes
- No

14- Have you ever seen foam in your urine after performing a high intensity physical effort?

- Yes
- No

15- Do you usually consume non-steroidal anti-inflammatory drugs (ibuprofen, spidifen, diclofenac, dexketoprofen, voltaren, enantyum, aspirin, acetylsalicylic acid, airtal)?

- Yes
- No
